# Outcome of In-Hospital Cardiac Arrest among Patients with COVID-19: A Systematic Review and Meta-Analysis

**DOI:** 10.3390/jcm12082796

**Published:** 2023-04-10

**Authors:** Dhan Bahadur Shrestha, Yub Raj Sedhai, Sagun Dawadi, Bishal Dhakal, Jurgen Shtembari, Karan Singh, Roshan Acharya, Soney Basnyat, Irfan Waheed, Mohammad Saud Khan, Mohammed Kazimuddin, Nimesh K. Patel, Gautham Kalahasty, Prashant Dattatraya Bhave, Patrick Whalen, Ghanshyam Shantha

**Affiliations:** 1 Department of Internal Medicine, Mount Sinai Hospital, Chicago, IL 60608, USA; 2 Division of Pulmonary Disease and Critical Care, University of Kentucky College of Medicine-Bowling Green Campus, E 1st Ave, Bowling Green, KY 42101, USA; 3 Department of Internal Medicine, Nepalese Army Institute of Health Sciences, Kathmandu 44600, Nepal; 4 Division of Pulmonary Disease and Critical Care Medicine, Virginia Tech Carilion School of Medicine, Roanoke, VA 24014, USA; 5 Department of Internal Medicine, University of Kentucky College of Medicine-Bowling Green Campus, E 1st Ave, Bowling Green, KY 42101, USA; 6 Division of Cardiology, University of Kentucky College of Medicine-Bowling Green Campus, E 1st Ave, Bowling Green, KY 42101, USA; 7 Department of Internal Medicine, Division of Cardiology, Virginia Commonwealth University School of Medicine, Richmond, VA 23219, USA; 8 Department of Internal Medicine, Division of Electrophysiology, Atrium Health Wake Forest Baptist Medical Center, Medical Center Boulevard, Winston-Salem, NC 27157, USA

**Keywords:** COVID-19, in-hospital cardiac arrest, mortality

## Abstract

Background: Outcomes following in-hospital cardiac arrest (IHCA) in patients with COVID-19 have been reported by several small single-institutional studies; however, there are no large studies contrasting COVID-19 IHCA with non-COVID-19 IHCA. The objective of this study was to compare the outcomes following IHCA between COVID-19 and non-COVID-19 patients. Methods: We searched databases using predefined search terms and appropriate Boolean operators. All the relevant articles published till August 2022 were included in the analyses. The systematic review and meta-analysis were conducted as per Preferred Reporting Items for Systematic Reviews and Meta-analyses (PRISMA) guidelines. An odds ratio with a 95% confidence interval (CI) was used to measure effects. Results: Among 855 studies screened, 6 studies with 27,453 IHCA patients (63.84% male) with COVID-19 and 20,766 (59.7% male) without COVID-19 were included in the analysis. IHCA among patients with COVID-19 has lower odds of achieving return of spontaneous circulation (ROSC) (OR: 0.66, 95% CI: 0.62–0.70). Similarly, patients with COVID-19 have higher odds of 30-day mortality following IHCA (OR: 2.26, 95% CI: 2.08–2.45) and have 45% lower odds of cardiac arrest because of a shockable rhythm (OR: 0.55, 95% CI: 0.50–0.60) (9.59% vs. 16.39%). COVID-19 patients less commonly underwent targeted temperature management (TTM) or coronary angiography; however, they were more commonly intubated and on vasopressor therapy as compared to patients who did not have a COVID-19 infection. Conclusions: This meta-analysis showed that IHCA with COVID-19 has a higher mortality and lower rates of ROSC compared with non-COVID-19 IHCA. COVID-19 is an independent risk factor for poor outcomes in IHCA patients.

## 1. Introduction

COVID-19 has been linked to a risk of cardiac arrest, which can be fatal if not treated promptly [1,2,3]. There is limited understanding and a paucity of data on in-hospital cardiac arrest (IHCA) among patients infected with COVID-19. Data are largely based on single-center studies and registry data analyses. The exact mechanisms by which COVID-19 can lead to cardiac arrest are not fully understood, but several factors have been proposed. As we know, the virus can affect the heart muscle, which can cause myocarditis and can also trigger a systemic inflammatory response leading to coagulation abnormalities. However, hypoxemia associated with severe COVID-19 is the most putative mechanism put forward for cardiac arrest associated with COVID-19 [2]. Single-center studies have reported survival to discharge ranging from 0 to 3% [1,2,3]; however, a large multicenter study has reported a survival to discharge of up to 7% [4]. While the incidence of IHCA is higher in the intensive care units (ICUs), survival to discharge is reported as lower among non-ICU patients [5].

Clinical outcomes in COVID-19-related IHCA vary with the severity of disease, the extent of muti-organ failure, age, comorbidities, and the size and resources of the clinical center [4,5]. It is important to recognize the risk of cardiac arrest in COVID-19 patients and to provide prompt treatment, including cardiopulmonary resuscitation (CPR) and defibrillation, as needed, to improve outcomes. As such, outcomes in IHCA in the setting of COVID-19 are speculated to be poor; however, the comparative pooling of COVID-19-associated IHCA with IHCA not related to COVID-19 and its determinants has not been fully explored. Thus, in order to fully appraise the available data, we sought to perform this systematic review and meta-analysis.

## 2. Materials and Methods

We used the Preferred Reporting Items for Systematic Review and Meta-Analysis (PRISMA) guidelines [6]. The study protocol was registered in the international prospective register of systematic reviews (PROSPERO ID: CRD42022351507) [7]. The participant, intervention, control, and outcome (PICO) framework was employed to formulate the review questions. A MOOSE (Meta-analyses Of Observational Studies in Epidemiology) [8] checklist is provided in the Supplementary Material.

### 2.1. Criteria for Considering Studies for This Review

#### 2.1.1. Types of Studies

Observational studies comparing the clinical outcomes of IHCA among patients with and without COVID-19 were included in this review. Non-comparative studies reporting IHCA without COVID-19 and studies from the pre-COVID-19 era were excluded. Similarly, viewpoints, case reports, case series, conference proceedings, editorials, and comments were excluded.

#### 2.1.2. Type of Participants

Patients older than 18 years sustaining IHCA in the setting of COVID-19 were considered participants, comprising the study arm. COVID-19 was diagnosed using a standard polymerase chain reaction (PCR)-based diagnostic test. Cardiac arrest patients without COVID-19 were considered comparators, comprising the control arm.

#### 2.1.3. Outcomes

The impact of the factors affecting clinical outcomes in cardiac arrest including age, initial rhythm, and comorbidities were compared between the study and the control arms. The primary outcomes of interest were the return of spontaneous circulation (ROSC) and 30-day mortality. Secondary outcomes of interest were the initial rhythm at the time of cardiac arrest, targeted temperature management (TTM), coronary angiography, need for vasopressor support, duration of mechanical ventilation, and renal replacement therapy (RRT).

By definition, pulseless ventricular tachycardia and ventricular fibrillation are considered shockable rhythm and pulseless electrical activity and asystole non-shockable rhythm. In all comatose cardiac arrest survivors, hypothermia protocol was followed to maintain a low core body temperature to avoid/minimize ongoing neurological damage secondary to cardiac arrest insult based on the current guidelines. Post-cardiac-arrest care also includes appropriate oxygenation and blood pressure maintenance with intravenous fluid and vasopressor use to optimize the perfusion of the vital organs.

### 2.2. Search Methods for Identification of Studies

We performed an extensive literature search in PubMed, Scopus, Cochrane Library, and Embase. We have included relevant studies that have compared IHCA among patients with and without COVID-19. Relevant articles published till August 2022 were included in the analyses.

#### Electronic Searches

The detailed search strategy has been attached in Supplementary Material S1.

### 2.3. Data Analysis

The extracted data were analyzed using Cochrane Review Manager (RevMan) version 5.4 [9]. Outcomes were measured using a fixed or random effect model for dichotomous outcomes and the mean difference (MD) for continuous outcomes.

#### 2.3.1. Selection, Data Extraction, and Management of Studies

Covidence systematic review software was used to screen studies [10]. Title, abstract, and full-text screening was performed independently by two reviewers and conflicts were resolved by a third reviewer. After the full-text review, relevant data from the included studies were extracted into Microsoft Excel by two reviewers (SD, BD) and the discrepancies were resolved by the third reviewer (DBS) and later analyzed. The assessment of the quality of the included studies was independently performed by two reviewers.

#### 2.3.2. Assessment of Risk of Bias in Included Studies

The Joanna Briggs Institute (JBI) critical appraisal tool was used for the assessment of the risk of bias [11]. A summary of the risk of bias is presented in Table 1.

#### 2.3.3. Assessment of Heterogeneity and Sensitivity Analysis

The heterogeneity in the included studies was determined using the I^2^ test using the *Cochrane Handbook for Systematic Reviews of Interventions* [18]. Heterogeneity above 40% was considered significant and a random effect model was applied. Further, sensitivity analysis was performed excluding outliers (studies with sample sizes of more than 5000 or less than 50).

#### 2.3.4. Assessment of Reporting Biases

Reporting bias was checked by prefixed reporting of the outcome.

#### 2.3.5. Data Synthesis

The Mantel–Haenszel method was used for the analysis of dichotomous outcomes. The effect size is measured using an odds ratio (OR) with a 95% confidence interval employing a fixed or random effect model depending on the heterogeneity of the data. Similarly, the inverse variance method was used for analyzing continuous outcomes. The mean difference was used as the measure of effect in fixed or random effect models, depending on the heterogeneity of the data under consideration.

## 3. Results

### 3.1. Qualitative Synthesis

**Figure 1:**
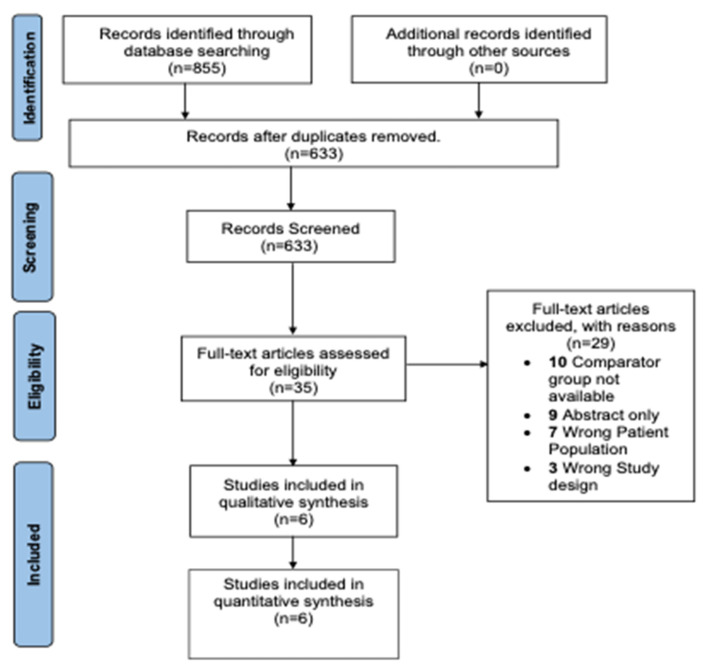
Preferred Reporting Items for Systematic Reviews and Meta-Analyses flow diagram.

A total of 855 studies were found from the database search. After removing duplicates, 633 studies were subjected to title and abstract screening where a total of 598 irrelevant studies were excluded. The full text of 35 studies was retrieved and comprehensively reviewed. Six observational studies were included in the qualitative and quantitative synthesis in the meta-analysis (Table 2 and Table 3). The details are presented in the PRISMA flow diagram presented in Figure 1.

The patients included in all the forementioned studies were followed for at least1 month in average to report 30-day mortality.

Girotra et al. [12] was the largest (*n* = 24,915) among all the included studies. This study compared the survival to discharge and ROSC for 20 min between COVID-19 and non-COVID-19 IHCA groups. In the study, patients with COVID-19 had lower rates of survival to discharge and ROSC. In the study by Holm et al. [14], both the Kaplan–Meir survival curve and odds ratio showed lower 30-day survival for COVID-19 with IHCA.

The study by Yuriditsky et al. [13] was a retrospective observational study conducted in a single center. The primary endpoint was ROSC, while 30-day survival and a cerebral performance category (CPC) of 1 or 2 were the secondary outcomes. Although the non-COVID-19 patients with IHCA had a better rate of ROSC and 30-day survival than the COVID-19 patients, it was not statistically significant.

Sultanian et al. [15] included both in- and out-hospital cardiac arrest before and during the pandemic in the study. However, the adjusted 30-day survival was lower in the COVID-19 patients as compared with non-COVID-19 patients with IHCA. The hazard ratio for death and the odds ratio for 30-day mortality were higher and the odds for ROSC were lower for the COVID-19 groups.

Aldabagh et al. [16] used a scoring system to evaluate the survival to discharge between the COVID-19 and non-COVID-19 groups. It was statistically lower in the COVID-19 groups. In the study by Roedl et al. [17], the Horowitz index (PaO_2_/FiO_2_) and resuscitation time were significantly lower in the COVID-19 group.

### 3.2. Quantitative Synthesis

A total of 6 studies with 27,453 IHCA patients were included in our analysis. Among them, 6687 patients sustained cardiac arrest in the setting of COVID-19 infection and 20,766 were non-COVID-19 cardiac arrest. Proportions of 64% among COVID-19 patients and 59.7% among non-COVID-19 patients were male.

A. Age

Five studies reported age as a continuous variable. Moreover, 3 of the 5 studies reported age in the mean with SD, while 2 of them [13,17] reported in the median with IQR. The mean was calculated from these 2 studies, employing the technique explained by Luo et al. and Xao et al. [19,20]. The average age among COVID-19 patients was 67.84 years while it was 69.34 years among non-COVID-19 patients. There was no significant mean difference in the age (MD = 0.00; 95% CI = −1.28 to 1.28; *n* = 2538; I^2^ = 0%) (Supplementary Material S2, Figure S1).

B. Comorbidities

The majority of the patients with IHCA had a prior history of medical comorbidities including but not limited to diabetes mellitus (DM), myocardial infarction (MI), congestive heart failure (CHF), and cerebrovascular accident (CVA). Proportions of 44.02% (2944/6687) of patients in the COVID-19 group and 34.54% (7173/20,766) among the non-COVID-19 group were diabetic (*p* > 0.05). Proportions of 9.97% (615/6170) of patients in the COVID-19 group and 14.94% (3039/20,346) in the non-COVID-19 group had a prior history of MI (*p* < 0.001) [13,15,16]. History of CHF was present among 18.09% (1116/6170) in the COVID-19 group compared to 24.16% (4915/20,346) in the non-COVID-19 group (*p* < 0.001) [13,15,16].

Three studies reported acute MI on admission, and 7.67% (473/6170) among COVID-19 patients and 13.83% (2814/20,346) among non-COVID-19 patients had acute MI on admission (*p* < 0.001). Acute CVA on admission was reported in 2.66% (164/6170) and 4.01% (815/20,346) of patients in the COVID-19 and non-COVID-19 groups (*p* < 0.001), respectively, as reported by three studies [13,15,16].

C. Shockable rhythm

**Figure 2:**
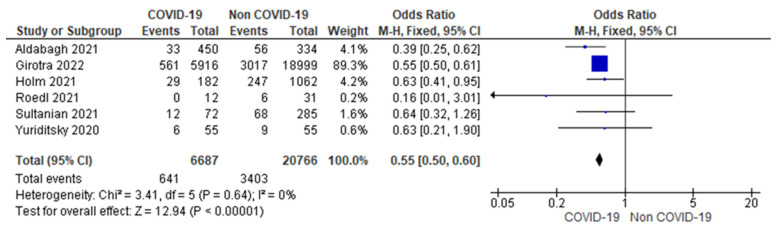
Forest plot showing group difference for shockable rhythm among COVID-19 versus non-COVID-19 patients using fixed effect. [12,13,14,15,16,17].

Meta-analysis of six studies showed 9.59% (641/6687) of COVID-19 patients and 16.39% (3403/20,766) of non-COVID-19 patients had an initial shockable rhythm (defined as pulseless ventricular tachycardia or ventricular fibrillation) at the time of cardiac arrest. There were statistically lower odds (45%) of an initial shockable rhythm in COVID-19-related cardiac arrest. (OR = 0.55; 95% CI = 0.50 to 0.60; *n* = 274,537; I^2^ = 0%) (Figure 2).

A sensitivity analysis was performed excluding the studies with more than 5000 or less than 50 sample sizes in each group [12,17]. Results were consistent with 43% lower odds of initial shockable rhythm in the COVID-19 group (OR = 0.53; 95% CI = 0.40 to 0.70; *n* = 2495; I^2^ = 0%) (Supplementary Material S2; Figure S2).

D. Return of spontaneous circulation (ROSC)

**Figure 3:**
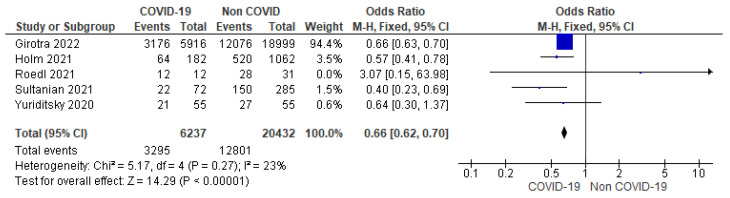
Forest plot showing group differences for ROSC among COVID-19 versus non-COVID-19 patients using fixed effect model. [12,13,14,15,17].

ROSC following cardiac arrest was reported in five studies. ROSC was achieved in 52.83% (3295/6237) of the patients in the COVID-19 group and 62.65% (12,801/20,432) in the non-COVID-19 group. There were significantly lower odds (34%) of ROSC in the COVID-19 group (OR = 0.66, 95% CI = 0.62 to 0.70; *n* = 26,669; I^2^ = 23%) (Figure 3).

Further, analysis excluding studies with sample sizes more than 5000 or less than 50 [12,17] showed a significant difference between the two groups (OR = 0.53; 95% CI = 0.41 to 0.69; *n* = 1711; I^2^ = 0%) (Supplementary Material S2; Figure S3).

E. Targeted temperature management (TTM)

**Figure 4:**
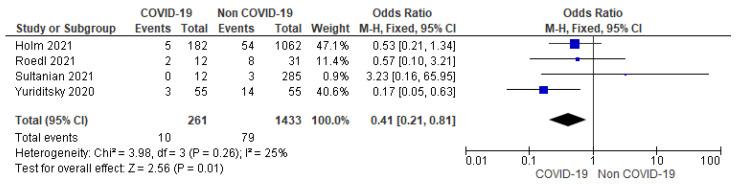
Forest plot showing group differences on TTM use among COVID-19 versus non-COVID-19 patients using fixed effect model. [13,14,15,17].

Four studies reported TTM status among the included studies. TTM was used in 3.83% (10/261) of COVID-19 patients and 5.51% (79/1433) of non-COVID-19 patients with significantly lower odds (59%) of TTM use among COVID-19 patients (OR = 0.41; 95% CI = 0.21 to 0.81; *n* = 1694; I^2^ = 25%) (Figure 4).

F. Coronary angiography

**Figure 5:**
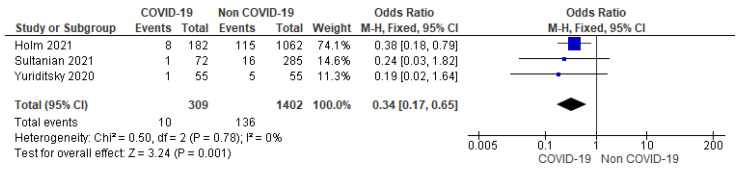
Forest plot showing group differences on coronary angiogram among COVID-19 versus non-COVID-19 patients using fixed effect model. [13,14,15].

Three studies reported the results of coronary angiography performed after cardiac arrest. Proportions of 3.24% (10/309) of COVID-19 patients and 9.7% (136/1402) of non-COVID-19 patients underwent coronary angiography following cardiac arrest with significantly lower odds of coronary angiography among COVID-19 patients (OR = 0.34; 95% CI = 0.17 to 0.65; *n* = 1711; I^2^ = 0%) (Figure 5).

G. Thirty-day mortality

**Figure 6:**
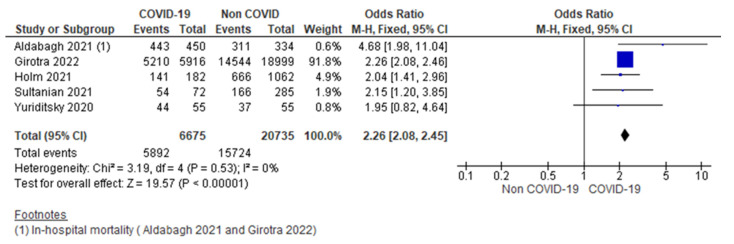
Forest plot showing group differences on 30-day mortality among COVID-19 versus non-COVID-19 patients using fixed effect model. [12,13,14,15,16].

Five studies reported 30-day mortality, showing 88.27% (5892/6675) among the COVID-19 group and 75.83% (15,724/20,735) in the non-COVID-19 group. COVID-19 patients sustaining cardiac arrest had 2.26 higher odds of having 30 days compared to the control group (OR = 2.26; 95% CI = 2.08 to 2.45; *n* = 27,410; I^2^ = 0%) (Figure 6).

Excluding an outlier study [12], also consistently showed a similar result (OR = 2.26; 95% CI = 1.71 to 2.98; *n* = 2495; I^2^ = 6%) (Supplementary Material S2: Figure S4).

H. Post-cardiac-arrest care

Vasopressor use

Two studies reported vasopressor use following cardiac arrest. Pooled analysis showed 1.53 times higher odds of requiring vasopressor support following cardiac arrest (OR = 1.53; 95% CI = 1.44 to 1.63; *n* = 24,958; I^2^ = 0%) (Supplementary Material S2: Figure S5).

Intubation

Two studies reported intubation following cardiac arrest, and a pooling of their data showed COVID-19 patients following cardiac arrest had 1.5 times higher odds of intubation (OR = 1.50, 95% CI = 1.14 to 1.96; *n* = 1601; I^2^ = 0%) (Supplementary Material S2: Figure S6).

Mechanical ventilator (MV)

There was no significant between-group difference of MV use following cardiac arrest (OR = 0.96; 95% CI = 0.18 to 5.12; *n* = 25,272; I^2^ = 97%) (Supplementary Material S2: Figure S7).

Renal replacement therapy (RRT)

Three studies reported post-arrest RRT. Pooled analysis showed COVID-19 patients following cardiac arrest had 1.33 higher odds of requiring RRT (OR = 1.33; 95% CI = 0.95 to 1.85; *n* = 25,742; I^2^ = 59%) (Supplementary Material S2: Figure S8).

I. Publication bias

Funnel plots were used to estimate publication bias. For both ROSC and 30-day mortality outcomes, funnel plots showed a nearly symmetric distribution of studies suggesting low publication bias across the included studies (Supplementary Material S2, Figures S9 and S10).

## 4. Discussion

In this meta-analysis, we sought to compare the outcomes of in-hospital cardiac arrest (IHCA) among patients with and without COVID-19 infection. We found that there was a significant difference in the rate of return of spontaneous circulation and mortality between the two groups.

Among the studies reporting ROSC as a primary outcome [12,13,14,15,17], there was a statistically significant lower rate of ROSC among COVID-19 patients.

Similar to our finding, in a meta-analysis by Bielski et al., subgroup analysis comparing ROSC between COVID-19 and non-COVID-19 patients found lower rate of ROSC in the COVID-19 population (33.9%) as compared to the non-COVID-19 patients (52.1%) [21]. A proportional prevalence meta-analysis including 4 studies and 943 IHCA patients with COVID-19 by Mir et al. [22] reported a lower pooled prevalence of ROSC (39%) than our study (52.83%).

Pooled analysis from five studies [12,13,14,15,16] showed 30-day mortality or in-hospital mortality following IHCA was 88.27% among the COVID-19 population. COVID-19 patients with cardiac arrest in the hospital had 2.26 higher odds of having 30-day or in-hospital mortality outcomes as compared to non-COVID-19 patients. This estimate of the odds of dying is similar to the observation by Ippolito et al. [23], which reported 2.34 times higher odds of dying in COVID-19 patients. This analysis included three observational studies [13,15,24].

Based on the observations from mortality outcomes, we made the initial assumption that different patient characteristics among the study population (COVID-19 vs. non-COVID-19) may be one of the reasons for this significant difference. We compared the reported patients’ characteristics to identify the differences between the two groups. There was no significant statistical difference in age and history of diabetes between the two groups; however, the observations from the included studies showed that non-COVID-19 patients had higher rates of comorbidities, such as MI on admission, a past history of MI, a history of heart failure, and CVA (*p* < 0.001). This observation points towards the fact that COVID-19 itself may be associated with poor outcomes following IHCA irrespective of the patient characteristics and comorbidities.

AHA in 2020 recommended targeted temperature management (TTM) of 32–34 degrees Celsius as a class I recommendation with a variable level of evidence in IHCA survivors with suspected brain injury. Thus, TTM has been established as an important post-cardiac-arrest intervention in individuals with suspected anoxic brain injury and has been compounded into guidelines [25,26]. Analysis of four studies reporting TTM showed significantly lower odds (59%) of TTM use among COVID-19 patients. This may be related to the fact that there is a paucity of information on TTM use among COVID-19 patients following cardiac arrest and further study is required in this regard [27].

Reports on the benefit of coronary angiography on the clinical outcomes from observational studies are inconsistent and a multicenter trial showed no significant effect on the outcomes [28]. Our study showed lower odds (66%) of pursuing emergent coronary angiography in COVID-19 patients as compared to the non-COVID-19 patients. This difference can be explained by the nature of cardiac arrest and initial rhythm among the COVID-19 population, and because of a weak level of evidence on the utility of emergent coronary angiography among COVID-19 patients. Another explanation may be remotely attributed to the fact from our previous observation that the non-COVID-19 population had a higher prevalence of MI on admission.

As previously mentioned, the pooled data from six included studies showed significantly lower odds (45%) of an initial shockable rhythm in COVID-19-related cardiac arrest. Subgroup analysis from an earlier meta-analysis showed similar odds of shockable rhythm in COVID-19 as compared to non-COVID-19 patients (OR: 0.51; 95% CI: 0.35–0.73; *p* < 0.001) [21]. Previous studies have shown that in patients with IHCA, shockable initial rhythm is associated with better clinical outcomes [29,30,31]. Estimation and assertion of this observation to the poor survival outcomes among COVID-19, however, requires further investigation. An increased delay to deliver defibrillation or start resuscitation was observed in the COVID-19 group. In Girotra et al., the systemic delays may have impacted the rate of successful resuscitation in patients with COVID-19.

Similarly, the pooled data from two included studies reporting intubation following IHCA showed COVID-19 patients following cardiac arrest had 1.5 times higher odds of intubation. COVID-19 is primarily a respiratory disease, with the lungs being a major affected organ. The significant impact of COVID-19 and the response to the disease lead to ARDS [32], which itself can be used to explain the higher rate of intubation in this subset of patients.

Our study has a few limitations given its statistical nature. First of all, the number of included studies is low and the included studies have their own limitations. A major limitation is the reported comorbidities among the individual studies. Furthermore, they were conducted in different parts of the world with different study populations that have their unique clinical characteristics. These limitations may affect the overall estimates of a particular outcome. Similarly, only retrospective studies were included in the review. Moreover, all the studies under consideration in our meta-analysis included COVID-19 patients with IHCA during the first year since the pandemic began and thus may not be representative of the overall COVID-19 pandemic, which is still ongoing. Lastly, the variant of the COVID-19 virus associated with IHCA has not been reported in the studies above. It is possible that different variants could have a different risk profile in terms of the mechanism and outcomes after cardiac arrest.

## 5. Conclusions

COVID-19 individuals have lower odds of ROSC and higher odds of 30-day mortality compared to non-COVID-19 individuals following IHCA. Considering the results of our study, we can make a preliminary assumption that COVID-19 is an independent predictor of ROSC and 30-day/in-hospital mortality. Moreover, established treatment modalities, such as TTM, are yet to be validated in the COVID-19 population.

## Figures and Tables

**Table 1:** Critical appraisal tool for the assessment of the risk of bias.

		Girotra et al., 2022 [12]	Yuriditsky et al., 2020 [13]	Holm et al., 2021 [14]	Sultanian et al., 2021 [15]	Aldabagh et al., 2021 [16]	Roedl et al., 2021 [17]
1	Were the two groups similar and recruited from the same population?	Yes	Yes	Yes	Yes	Yes	Yes
2	Were the exposures measured similarly to assign people to both exposed and unexposed groups?	Yes	Yes	Yes	Yes	Yes	Yes
3	Was the exposure measured in a valid and reliable way?	Yes	Yes	Yes	Yes	Yes	Yes
4	Were confounding factors identified?	Yes	Yes	No	No	Yes	Yes
5	Were strategies to deal with confounding factors stated?	No	No	NA	NA	Yes	No
6	Were the groups/participants free of the outcome at the start of the study (or at the moment of exposure)?	Yes	Yes	Yes	Yes	Yes	Yes
7	Were the outcomes measured in a valid and reliable way?	Yes	Yes	Yes	Yes	Yes	Yes
8	Was the follow up time reported and sufficient to be long enough for outcomes to occur?	Yes	Yes	Yes	Yes	Yes	Yes
9	Was follow up complete, and if not, were the reasons to loss to follow up described and explored?	Yes	Yes	Yes	Yes	Yes	Yes
10	Were strategies to address incomplete follow up utilized?	NA	NA	NA	NA	NA	NA
11	Was appropriate statistical analysis used?	Yes	Yes	Yes	Yes	Yes	Yes
Overall appraisal		Include	Include	Include	Include	Include	Include

**Table 2:** Baseline characteristics of included studies.

Study	Country	Type of Study	Total No of Participants (N)			Male	Age in Years, Mean (SD)	Initial Rhythm	
								Non-Shockable	Shockable
Girotra et al., 2022 [12]	United States	Cohort Study	24,915	With COVID-19	5916/24,915	3778/5916		5355/5916	561/5916
				Without COVID-19	18,999/24,915	11,288/18,999		15,982/18,999	3017/18,999
Yuriditsky et al., 2020 [13]	United States	Observational Study	110	With COVID-19	55/110	48/55	70.06 (9.896)	49/55	6/55
				Without COVID-19	55/110	33/55	68.82 (15.60)	46/55	9/55
Holm et al., 2021 [14]	Sweden	Observational Study	1613	With COVID-19	182/1613	114/182	70.93 (12.43)	153/182	29/182
				Without COVID-19	1062/1613	674/1062	71.00 (13.32)	815/1062	247/1062
Sultanian et al., 2021 [15]	Sweden	Cohort Study	1080	With COVID-19	72/1080	49/72	67.8 (13.0)	60/72	12/72
				Without COVID-19	285/1080	192/285	67.0 (20.8)	217/285	68/285
Aldabagh et al., 2021 [16]	United States	Observational Study	784	With COVID-19	450/784	271/450	66.4 (13.1)	370/450	33/450
				Without COVID-19	334/784	186/334	66.8 (15.5)	277/334	56/334
Roedl et al., 2021 [17]	Germany	Cohort Study	43	With COVID-19	12/43	9/12	65 (15.09)	12/12	0
				Without COVID-19	31/43	25/31	63.93 (20.98)	25/31	6/31

**Table 3:** Comorbidities and outcome of included studies.

Study		Comorbidities					Post-IHCA Procedures		Cardiac Arrest Survival Outcomes	
		DM	CAD	History of MI	MI at Admission	History of HF	TTM	Emergent Angiography	ROSC	30-Day Mortality
Girotra et al., 2022 [12]	With COVID-19	2616/5916		599/5916	460/5916	1074/5916			3176/5916	
	Without COVID-19	6761/18,999		2856/18,999	2613/18,999	4648/18,999			12,076/18,999	
Yuriditsky et al., 2020 [13]	With COVID-19	17/55	8/55				3/55	1/55	21/55	44/55
	Without COVID-19	23/55	33/55				14/55	5/55	27/55	37/55
Holm et al., 2021 [14]	With COVID-19	36/182		13/182	12/182	36/182	5/182	8/182	64/182	141/182
	Without COVID-19	224/1062		163/1062	178/1062	229/1062	54/1062	115/1062	520/1062	666/1062
Sultanian et al., 2021 [15]	With COVID-19	11/72		3/72	1/72	6/72	0	1/72	22/72	54/72
	Without COVID-19	28/285		20/285	23/285	38/285	3/285	16/285	150/285	166/285
Aldabagh et al., 2021 [16]	With COVID-19	260/450	97/450							
	Without COVID-19	128/334	116/334							
Roedl et al., 2021 [17]	With COVID-19						2/12		12/12	
	Without COVID-19						8/31		25/31	

Abbreviations: CAD: coronary artery disease; DM: Diabetes mellitus; HF: Heart failure; IHCA: In-hospital cardiac arrest; MI: Myocardial infarction; ROSC: Return of spontaneous circulation; SD: Standard deviation; TTM: targeted temperature management.

## Data Availability

As this is meta-analysis of published data, we provided the analyzed data in the manuscript and supplement.

## Supplementary Materials

The following supporting information can be downloaded at: https://www.mdpi.com/article/10.3390/jcm12082796/s1.
