# Supplementary material for: Outcome of In-Hospital Cardiac Arrest among Patients with COVID-19: A Systematic Review and Meta-Analysis
Source: J Clin Med. 2023 Apr 10;12(8):2796. doi: 10.3390/jcm12082796 (PMC10144838; doi:10.3390/jcm12082796)
Supplement: Supplementary file 1 [file jcm-12-02796-s001.zip › jcm-2250817-supplementary.pdf]

**Supplementary materials**

# Outcome of In-Hospital Cardiac Arrest in COVID-19 Patient: A Systematic Review and Meta-Analysis

**Dhan Bahadur Shrestha[1*], Yub Raj Sedhai[2], Sagun Dawadi[3], Bishal Dhakal[3], Jurgen Shtembari[1], Karan Singh[2], Roshan Acharya[2], Soney Basnyat[5], Irfan Waheed[2], Mohammad Saud Khan[6], Mohammad Kazimuddin[6], Nimesh Kirit Patel[7], Gautham Kalahasty[7], Prashant D. Bhave[8], S. Patrick Whalen[8], Ghanshyam Shantha[8*]**

1 Department of Internal Medicine, Mount Sinai Hospital, Chicago, IL 60608, USA
2 Department of Internal Medicine, Division of Pulmonary Disease and Critical Care, University of Kentucky College of Medicine-Bowling Green Campus, E 1st Ave, Bowling Green, KY 42101, USA
3 Department of Internal Medicine, Nepalese army institute of health sciences, Kathmandu, Nepal 44600
4 Division of Pulmonary Disease and Critical Care Medicine, Virginia Tech Carilion School of Medicine, Roanoke, VA, 24014
5 Department of Internal Medicine, University of Kentucky College of Medicine-Bowling Green Campus, E 1st Ave, Bowling Green, KY 42101, USA
6 Division of Cardiology, University of Kentucky College of Medicine-Bowling Green Campus, E 1st Ave, Bowling Green, KY 42101, USA
7 Department of Internal Medicine, Division of Cardiology, Virginia Commonwealth University, School of Medicine, Richmond, VA 23219, USA
8 Department of Internal Medicine, Division of Electrophysiology, Atrium Health Wake Forest Baptist Medical Center, Medical Center Boulevard, Winston-Salem, NC 27157

**Supplementary Material S1. COVID-19 IHCA search details**

PubMed

Search: ((COVID-19) OR (Coronavirus disease) OR (SARS-CoV-2) OR (severe acute respiratory syndrome coronavirus 2)) AND ((In-hospital Cardiac Arrest) OR IHCA)

Hits: 541

Link: https://pubmed.ncbi.nlm.nih.gov/?term=%28%28COVID-19%29+OR+%28Coronavirus+disease%29+OR+%28SARS-CoV-2%29+OR+%28severe+acute+respiratory+syndrome+coronavirus+2%29%29+AND+%28%28In-hospital+Cardiac+Arrest%29+OR+IHCA%29&filter=years.2019-2022&sort=date

Embase

Search: ('covid 19'/exp OR 'covid 19' OR 'coronavirus disease' OR (('coronavirus'/exp OR coronavirus) AND ('disease'/exp OR disease)) OR 'sars cov 2'/exp OR 'sars cov 2' OR 'severe acute respiratory syndrome coronavirus 2'/exp OR 'severe acute respiratory syndrome coronavirus 2' OR (severe AND acute AND ('respiratory'/exp OR respiratory) AND ('syndrome'/exp OR syndrome) AND ('coronavirus'/exp OR coronavirus) AND ('2'/exp OR 2))) AND ('in-hospital cardiac arrest' OR ('in hospital' AND ('cardiac'/exp OR cardiac) AND ('arrest'/exp OR arrest)) OR ihca)

Hits: 183

Link:
https://www.embase.com/#advancedSearch/resultspage/history.1/page.1/25.items/orderby.date/source.

Scopus

Search: TITLE-ABS-KEY ( ( ( covid-19 ) OR ( coronavirus AND disease ) OR ( sars-cov-2 ) OR ( severe AND acute AND respiratory AND syndrome AND coronavirus 2 ) ) AND ( ( in-hospital AND cardiac AND arrest ) OR ihca ) )

Hits: 127

Link: https://www.scopus.com/results/results.uri?sort=plf-f&src=s&st1=%28%28COVID-19%29+OR+%28Coronavirus+disease%29+OR+%28SARS-CoV-2%29+OR+%28severe+acute+respiratory+syndrome+coronavirus+2%29%29+AND+%28%28In-hospital+Cardiac+Arrest%29+OR+IHCA%29&sid=6b128330d5732d9dc9c89dd7a6f2f8e3&sot=b&sdt=b&sl=164&s=TITLE-ABS-KEY%28%28%28COVID-19%29+OR+%28Coronavirus+disease%29+OR+%28SARS-CoV-2%29+OR+%28severe+acute+respiratory+syndrome+coronavirus+2%29%29+AND+%28%28In-hospital+Cardiac+Arrest%29+OR+IHCA%29%29&origin=searchbasic&editSaveSearch=&yearFrom=Before+1960&yearTo=Present&featureToggles=FEATURE_EXPORT_REDESIGN:0

Cochrane library

Search: ((COVID-19) OR (Coronavirus disease) OR (SARS-CoV-2) OR (severe acute respiratory syndrome coronavirus 2)) AND ((In-hospital Cardiac Arrest) OR IHCA)

Hits: 4

Link: https://www.cochranelibrary.com/advanced-search

Supplementary Material S2

| Study or Subgroup | COVID-19 Mean | SD | Total | Non COVID-19 Mean | SD | Total | Weight | Mean Difference IV, Fixed, 95% CI |
|---|---|---|---|---|---|---|---|---|
| Aldabagh 2021 | 66.4 | 13.1 | 450 | 66.8 | 15.5 | 334 | 38.8% | -0.40 [-2.46, 1.66] |
| Holm 2021 | 70.93 | 12.43 | 182 | 71 | 13.32 | 1062 | 42.0% | -0.07 [-2.05, 1.91] |
| Roedl 2021 | 65 | 15.09 | 12 | 63.93 | 20.98 | 31 | 1.3% | 1.07 [-10.22, 12.36] |
| Sultanian 2021 | 67.8 | 13 | 72 | 67 | 20.8 | 285 | 11.0% | 0.80 [-3.05, 4.65] |
| Yuriditsky 2020 | 70.06 | 9.896 | 55 | 68.82 | 15.6 | 55 | 6.9% | 1.24 [-3.64, 6.12] |
| **Total (95% CI)** | | | **771** | | | **1767** | **100.0%** | **0.00 [-1.28, 1.28]** |

Heterogeneity: Chi² = 0.60, df = 4 (P = 0.96); I² = 0%
Test for overall effect: Z = 0.00 (P = 1.00)

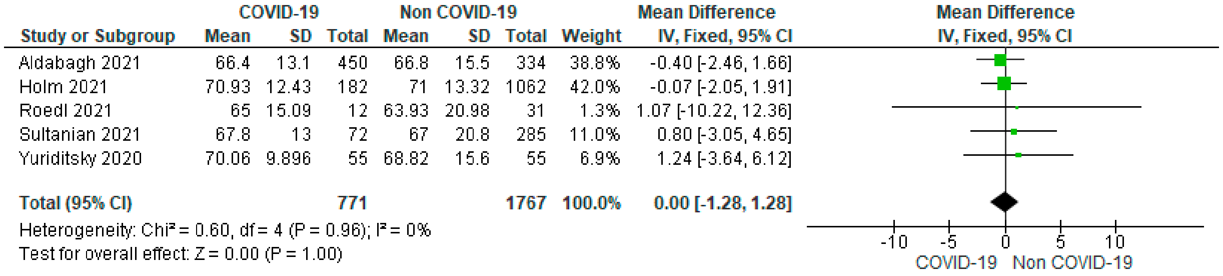


Supplementary material S2; Figure S1. Mean differences of age among patients across 5 studies between COVID-19 and non-COVID-19 patients using fixed effect model

| Study or Subgroup | COVID-19 Events | Total | Non COVID Events | Total | Weight | Odds Ratio M-H, Random, 95% CI |
|---|---|---|---|---|---|---|
| Aldabagh et al 2021 | 33 | 450 | 56 | 334 | 35.8% | 0.39 [0.25, 0.62] |
| Girotra et al 2022 | 561 | 5916 | 15982 | 18999 | 0.0% | 0.11 [0.10, 0.12] |
| Holm et al 2021 | 29 | 182 | 247 | 1062 | 41.9% | 0.63 [0.41, 0.95] |
| Pedram et al 2020 | 12 | 72 | 68 | 285 | 16.3% | 0.64 [0.32, 1.26] |
| Roedl et al 2021 | 0 | 12 | 6 | 31 | 0.0% | 0.19 [0.01, 3.12] |
| Yuriditsky et al 2020 | 6 | 55 | 9 | 55 | 6.1% | 0.63 [0.21, 1.90] |
| **Total (95% CI)** | | **759** | | **1736** | **100.0%** | **0.53 [0.40, 0.70]** |
| Total events | 80 | | 380 | | | |

Heterogeneity: Tau² = 0.00; Chi² = 2.63, df = 3 (P = 0.45); I² = 0%
Test for overall effect: Z = 4.54 (P < 0.00001)

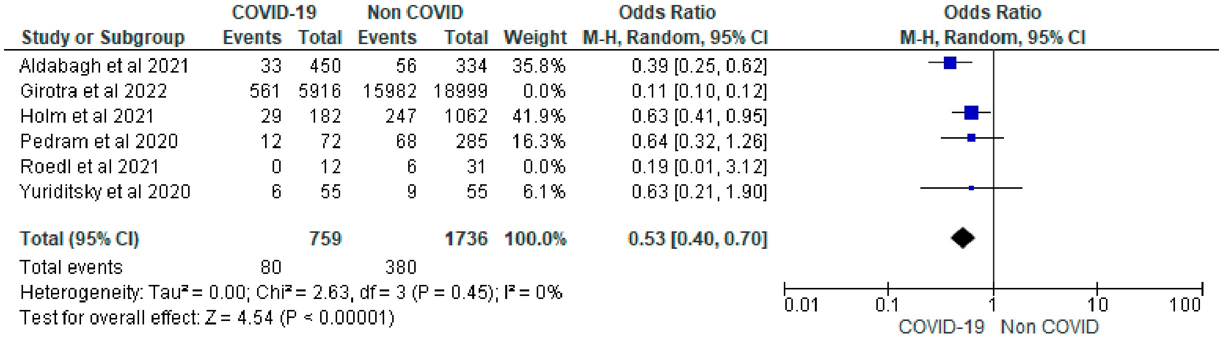


Supplementary material S2; Figure S2. Forest plot showing group difference for shockable rhythm among COVID-19 versus non-COVID-19 patient using fixed effect after excluding the outlier studies from analysis (Girotra *et al.,* Roedl *et al.*)

| Study or Subgroup | COVID-19 Events | Total | Non COVID Events | Total | Weight | Odds Ratio M-H, Fixed, 95% CI |
|---|---|---|---|---|---|---|
| Girotra 2022 | 3176 | 5916 | 12076 | 18999 | | Not estimable |
| Holm 2021 | 64 | 182 | 520 | 1062 | 62.7% | 0.57 [0.41, 0.78] |
| Roedl 2021 | 12 | 12 | 28 | 31 | | Not estimable |
| Sultanian 2021 | 22 | 72 | 150 | 285 | 26.7% | 0.40 [0.23, 0.69] |
| Yuriditsky 2020 | 21 | 55 | 27 | 55 | 10.6% | 0.64 [0.30, 1.37] |
| **Total (95% CI)** | | **309** | | **1402** | **100.0%** | **0.53 [0.41, 0.69]** |
| Total events | 107 | | 697 | | | |

Heterogeneity: Chi² = 1.46, df = 2 (P = 0.48); I² = 0%
Test for overall effect: Z = 4.75 (P < 0.00001)

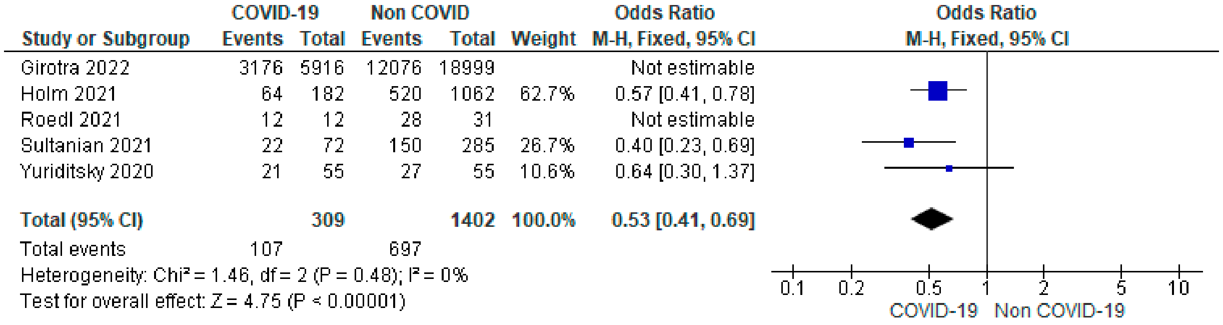


Supplementary material S2; Figure S3. Forest plot showing group differences for ROSC among COVID-19 versus non-COVID-19 patient using fixed effect model after removing outlier studies. (Girotra *et al.* and Roedl *et al.*)

| Study or Subgroup | COVID-19 Events | COVID-19 Total | Non COVID Events | Non COVID Total | Weight | Odds Ratio M-H, Fixed, 95% CI |
|---|---|---|---|---|---|---|
| Aldabagh 2021 (1) | 443 | 450 | 311 | 334 | 7.5% | 4.68 [1.98, 11.04] |
| Girotra 2022 | 5210 | 5916 | 14544 | 18999 | 0.0% | 2.26 [2.08, 2.46] |
| Holm 2021 | 141 | 182 | 666 | 1062 | 59.7% | 2.04 [1.41, 2.96] |
| Sultanian 2021 | 54 | 72 | 166 | 285 | 22.7% | 2.15 [1.20, 3.85] |
| Yuriditsky 2020 | 44 | 55 | 37 | 55 | 10.1% | 1.95 [0.82, 4.64] |
| **Total (95% CI)** | | **759** | | **1736** | **100.0%** | **2.26 [1.71, 2.98]** |
| Total events | 682 | | 1180 | | | |

Heterogeneity: Chi² = 3.19, df = 3 (P = 0.36); I² = 6%

Test for overall effect: Z = 5.76 (P < 0.00001)

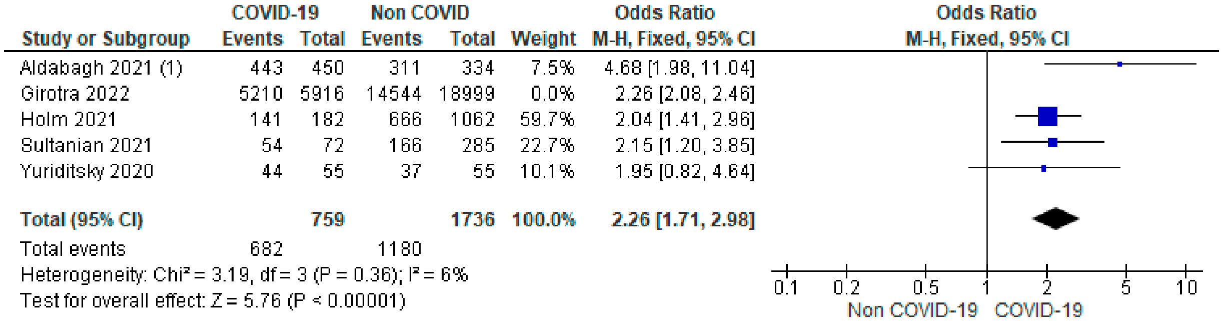


Footnotes

(1) In-hospital mortality ( Aldabagh 2021 and Girotra 2022)

Supplementary material S2: Figure S4. Excluding Girotra *et al* for 30-day mortality among COVID-19 versus non-COVID-19 patient using fixed effect model

| Study or Subgroup | COVID-19 Events | COVID-19 Total | Non COVID-19 Events | Non COVID-19 Total | Weight | Odds Ratio M-H, Fixed, 95% CI |
|---|---|---|---|---|---|---|
| Girotra 2022 | 2319 | 5916 | 5618 | 18999 | 99.9% | 1.54 [1.44, 1.63] |
| Roedl 2021 | 11 | 12 | 29 | 31 | 0.1% | 0.76 [0.06, 9.23] |
| **Total (95% CI)** | | **5928** | | **19030** | **100.0%** | **1.53 [1.44, 1.63]** |
| Total events | 2330 | | 5647 | | | |

Heterogeneity: Chi² = 0.31, df = 1 (P = 0.58); I² = 0%

Test for overall effect: Z = 13.82 (P < 0.00001)

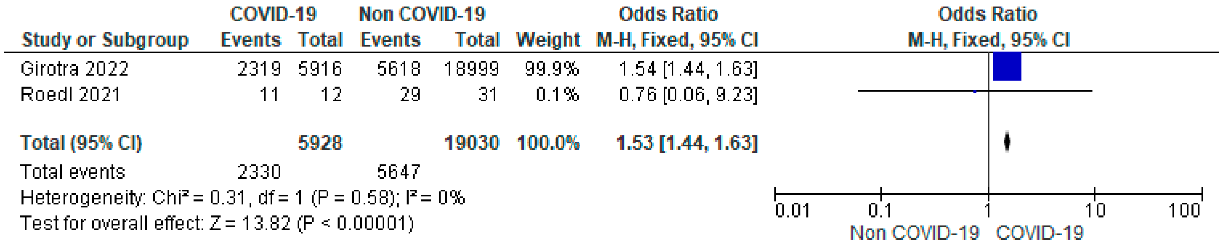


Supplementary material S2: Figure S5. Forest plot showing group differences on vasopressor use among COVID-19 versus non-COVID-19 patient using fixed effect model

| Study or Subgroup | COVID-19 Events | COVID-19 Total | Non COVID-19 Events | Non COVID-19 Total | Weight | Odds Ratio M-H, Fixed, 95% CI |
|---|---|---|---|---|---|---|
| Holm 2021 | 100 | 182 | 473 | 1062 | 72.7% | 1.52 [1.11, 2.08] |
| Sultanian 2021 | 35 | 72 | 113 | 285 | 27.3% | 1.44 [0.86, 2.42] |
| **Total (95% CI)** | | **254** | | **1347** | **100.0%** | **1.50 [1.14, 1.96]** |
| Total events | 135 | | 586 | | | |

Heterogeneity: Chi² = 0.03, df = 1 (P = 0.86); I² = 0%

Test for overall effect: Z = 2.93 (P = 0.003)

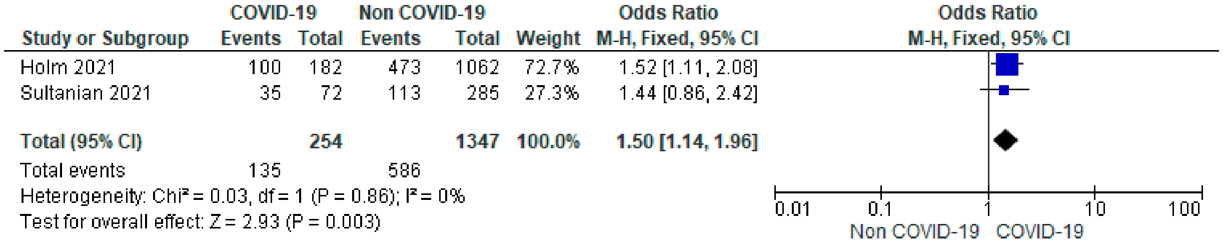


Supplementary material S2: Figure S6. Forest plot showing group differences on intubation among COVID-19 versus non-COVID-19 patient using fixed effect model

| Study or Subgroup | COVID-19 Events | COVID-19 Total | Non COVID-19 Events | Non COVID-19 Total | Weight | Odds Ratio M-H, Random, 95% CI |
|---|---|---|---|---|---|---|
| Girotra 2022 | 3412 | 5916 | 7265 | 18999 | 51.4% | 2.20 [2.07, 2.34] |
| Sultanian 2021 | 20 | 72 | 140 | 285 | 48.6% | 0.40 [0.23, 0.70] |
| **Total (95% CI)** | | **5988** | | **19284** | **100.0%** | **0.96 [0.18, 5.12]** |
| Total events | 3432 | | 7405 | | | |

Heterogeneity: $Tau^2 = 1.42$; $Chi^2 = 34.72$, df = 1 (P < 0.00001); $I^2 = 97\%$
Test for overall effect: Z = 0.05 (P = 0.96)

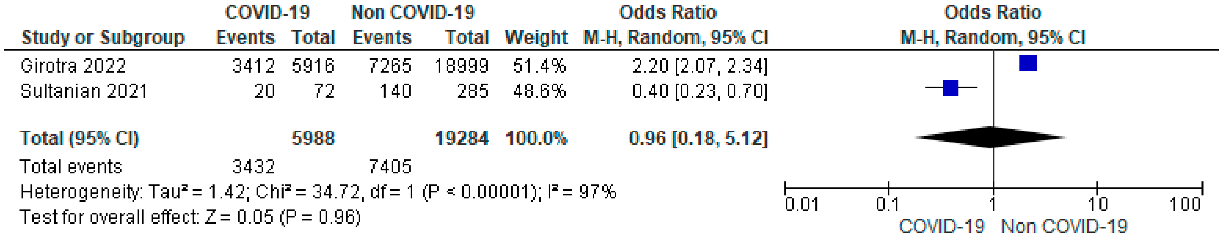


Supplementary material S2: Figure S7. Forest plot showing group difference on Mechanical Ventilation among COVID-19 versus non COVID-19 patient using Random effect model.

| Study or Subgroup | COVID-19 Events | COVID-19 Total | Non COVID-19 Events | Non COVID-19 Total | Weight | Odds Ratio M-H, Random, 95% CI |
|---|---|---|---|---|---|---|
| Aldabagh 2021 | 122 | 450 | 81 | 334 | 39.5% | 1.16 [0.84, 1.61] |
| Girotra 2022 | 280 | 5916 | 700 | 18999 | 56.8% | 1.30 [1.13, 1.50] |
| Roedl 2021 | 10 | 12 | 12 | 31 | 3.7% | 7.92 [1.47, 42.54] |
| **Total (95% CI)** | | **6378** | | **19364** | **100.0%** | **1.33 [0.95, 1.85]** |
| Total events | 412 | | 793 | | | |

Heterogeneity: $Tau^2 = 0.05$; $Chi^2 = 4.88$, df = 2 (P = 0.09); $I^2 = 59\%$
Test for overall effect: Z = 1.67 (P = 0.09)

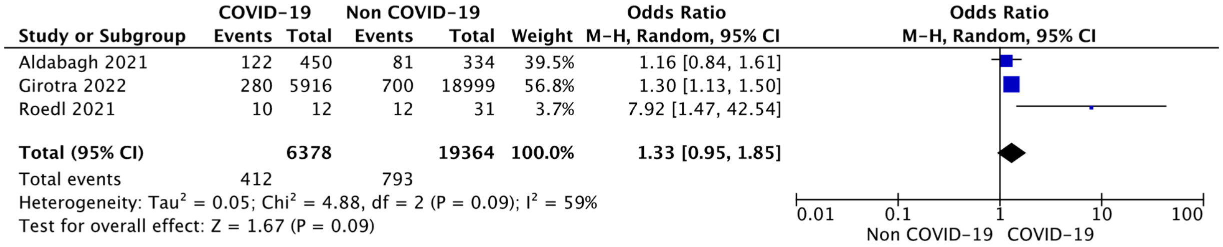


Supplementary material S2: Figure S8. Forest plot showing group difference on Renal Replacement Therapy among COVID-19 Versus non COVID-19 patient using Random effect model.

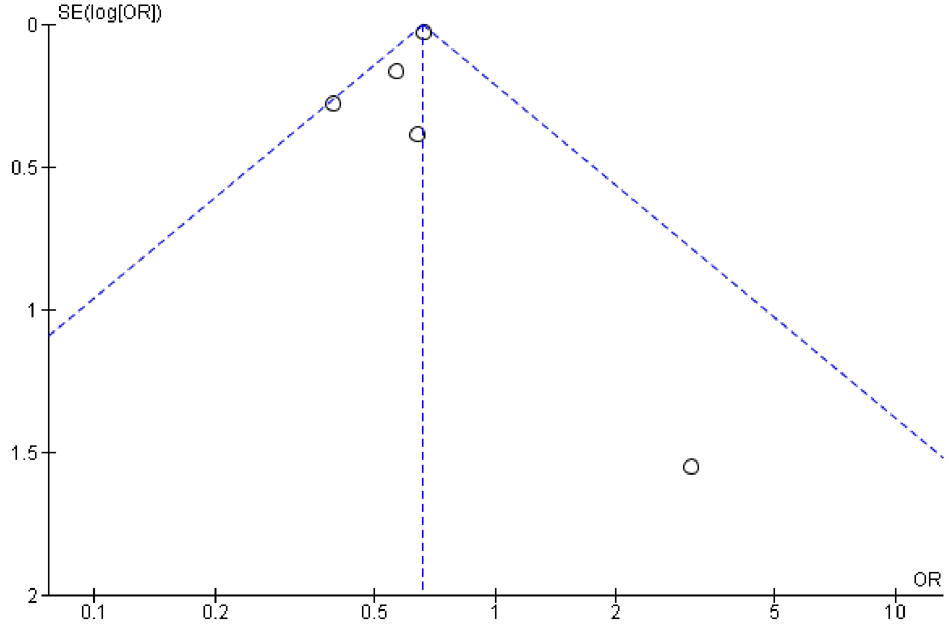


Supplementary material S2, Figure S9. Funnel plot showing publication bias for ROSC

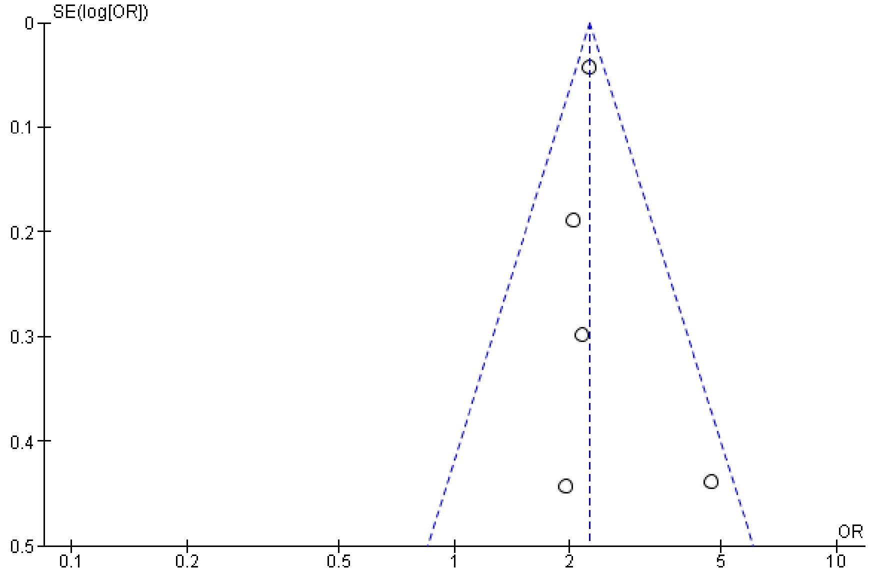


Supplementary material S2, Figure S10. Funnel plot showing publication bias for 30-day mortality

Supplementary Material S3. **MOOSE Checklist for Meta-analyses of Observational Studies**

| Item No | Recommendation | Reported on Page No |
|---|---|---|
| Reporting of background should include | | |
| 1 | Problem definition | 5 |
| 2 | Hypothesis statement | - |
| 3 | Description of study outcome(s) | 6 |
| 4 | Type of exposure or intervention used | 6 |
| 5 | Type of study designs used | 5 |
| 6 | Study population | 6 |
| Reporting of search strategy should include | | |
| 7 | Qualifications of searchers (eg, librarians and investigators) | 1 |
| 8 | Search strategy, including time period included in the synthesis and key words | 6, online supplementary material 1 |
| 9 | Effort to include all available studies, including contact with authors | 6 |
| 10 | Databases and registries searched | 6 |
| 11 | Search software used, name and version, including special features used (eg, explosion) | 6 |
| 12 | Use of hand searching (eg, reference lists of obtained articles) | - |
| 13 | List of citations located and those excluded, including justification | 8-9 |
| 14 | Method of addressing articles published in languages other than English | 7 |
| 15 | Method of handling abstracts and unpublished studies | 8-9 |
| 16 | Description of any contact with authors | - |
| Reporting of methods should include | | |
| 17 | Description of relevance or appropriateness of studies assembled for assessing the hypothesis to be tested | 7 |
| 18 | Rationale for the selection and coding of data (eg, sound clinical principles or convenience) | 7-8 |
| 19 | Documentation of how data were classified and coded (eg, multiple raters, blinding and interrater reliability) | 7-8 |
| 20 | Assessment of confounding (eg, comparability of cases and controls in studies where appropriate) | 7, supplementary material 2 |
| 21 | Assessment of study quality, including blinding of quality assessors, stratification or regression on possible predictors of study results | 7 |
| 22 | Assessment of heterogeneity | 7 |
| 23 | Description of statistical methods (eg, complete description of fixed or random effects models, justification of whether the chosen models account for predictors of study results, dose-response models, or cumulative meta-analysis) in sufficient detail to be replicated | 7-8 |

| | | |
|---|---|---|
| 24 | Provision of appropriate tables and graphics | 10-16 supplementary materials |
| Reporting of results should include | | |
| 25 | Graphic summarizing individual study estimates and overall estimate | Fig 2-3, supplementary material |
| 26 | Table giving descriptive information for each study included | Table 1 and 2 |
| 27 | Results of sensitivity testing (eg, subgroup analysis) | 10 – 16, Figs 2-3, supplementary material |
| 28 | Indication of statistical uncertainty of findings | - |

| **Item No** | **Recommendation** | **Reported on Page No** |
|---|---|---|
| Reporting of discussion should include | | |
| 29 | Quantitative assessment of bias (eg, publication bias) | 7, Table 1 in supplementary file |
| 30 | Justification for exclusion (eg, exclusion of non-English language citations) | 7 |
| 31 | Assessment of quality of included studies | 7 |
| Reporting of conclusions should include | | |
| 32 | Consideration of alternative explanations for observed results | 17-20 |
| 33 | Generalization of the conclusions (ie, appropriate for the data presented and within the domain of the literature review) | 19-20 |
| 34 | Guidelines for future research | 20 |
| 35 | Disclosure of funding source | 20 |

*From*: Stroup DF, Berlin JA, Morton SC, et al, for the Meta-analysis Of Observational Studies in Epidemiology (MOOSE) Group. Meta-analysis of Observational Studies in Epidemiology. A Proposal for Reporting. *JAMA*. 2000;283(15):2008-2012. doi: 10.1001/jama.283.15.2008.
